# A convenient and self-adjustable laparoscopic intracorporeal pringle maneuver using He’s belt

**DOI:** 10.1186/s12893-025-03214-y

**Published:** 2025-10-03

**Authors:** Huilan Zeng, Jiaqiang Mo, Sili Lu, Qing Ye, Zhangyuanzhu Liu, Jianyi Wang, Yanbei Yin, Peiying Zhong, Ting Xiao, Yanjiao Hu, Lijun Lin, Jianxin Peng, Junming He

**Affiliations:** Department of Hepatobiliary Surgery, Guangdong Provincial Hospital of Chinese Medicine, Guangzhou, 510120 People’s Republic of China

## Abstract

**Purpose:**

This study introduces an intracorporeal Pringle maneuver (PM) using He’s belt for laparoscopic hepatectomy.

**Methods:**

He’s belt, a silicone belt featuring a hole in the tail and eight cogs in the body, was placed in the abdominal cavity. The head and body of He’s belt were pulled out through the tail hole to form a ring encircling the hepatoduodenal ligament. We conducted laparoscopic hepatectomy with PM maneuver by He’s belt for 47 patients between January 2024 and June 2024.

**Result:**

The PM maneuver using He’s belt can be easily performed, adjusted, and released without requiring additional Hemoclips. For the 47 patients, the median operative time was 121 min. The median Pringle time was 21 min. The median blood loss was 50 ml. None of the patients required intraoperative blood transfusion.

**Conclusion:**

The PM maneuver using He’s belt is a safe, convenient, and self-adjustable method that provides effective inflow control for laparoscopic hepatectomy.

## Introduction

Compared to open hepatectomy (OH), laparoscopic hepatectomy (LH) offers advantages, including fewer perioperative complications and comparable overall survival rates. It is widely performed for patients with hepatolithiasis, benign liver tumors, primary liver cancer, or liver metastases [1, 2]. Excessive blood loss and subsequent perioperative blood transfusions are associated with increased morbidity and mortality [3, 4]. Effective techniques for controlling hepatic bleeding during LH are therefore critical to improving patient outcomes.

The Pringle maneuver (PM) is the first method developed to occlude the hepatic inflow, significantly reducing hepatic bleeding and providing a clear operative field during open hepatic transection [5]. With the increasing adoption of minimally invasive liver resection (MILR), various techniques for performing PM have been developed [6]. However, existing methods, such as the Rummel tourniquet, bulldog clamp, and Huang’s loop, have limitations, including the need for additional trocars, complex manipulations, or extra hemoclips [7–10]. 

This study introduces He’s belt, an innovative, and self-adjustable device designed to address these limitations. Based on Huang’s loop [7], He’s belt simplifies the PM procedure while maintaining efficacy and safety.

## Materials and methods

### The He’s belt

He’s belt, designed by the Department of Hepatobiliary Surgery at Guangdong Provincial Hospital of Chinese Medicine, with subsequent product development entrusted to Henan Tuoren Medical Technology Co., Ltd, has been patented (Patent No. ZL202220295632.5). To reduce blood loss and the need for perioperative blood transfusions, the He’s belt has been used routinely in our center since June 2023, especially for laparoscopic anatomic liver resection. For patients undergoing laparoscopic liver resection such as hemihepatectomy, lobe hepatectomy, segment hepatectomy, or non-anatomical hepatectomy, we clamped the hepatoduodenal ligament by using He’s belt.

**Fig. 1:**
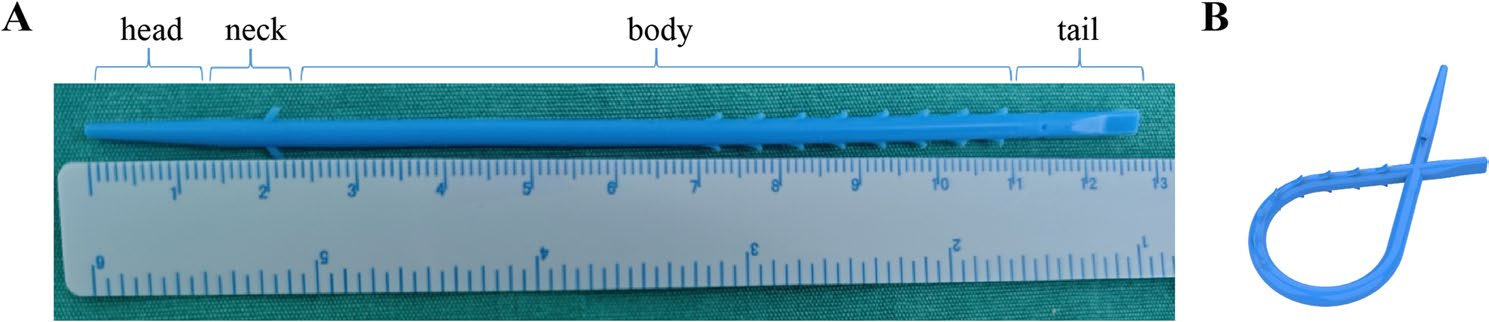
The introduction of He’s belt. **A** Basic sturcture of He’s belt. **B** Ring structure of He’s belt

**Fig. 2:**
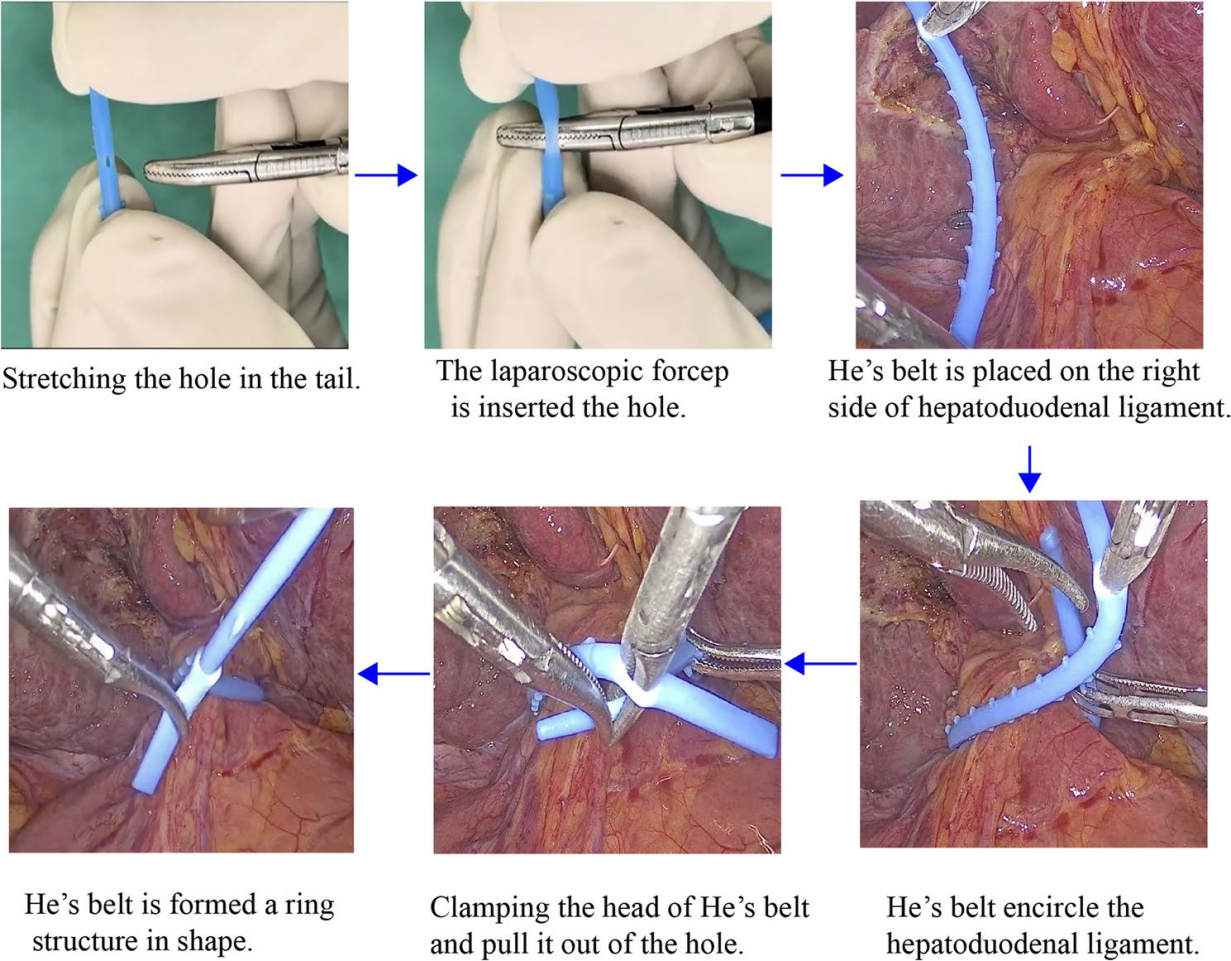
The procedure of presetting of He’s belt

**Fig. 3:**
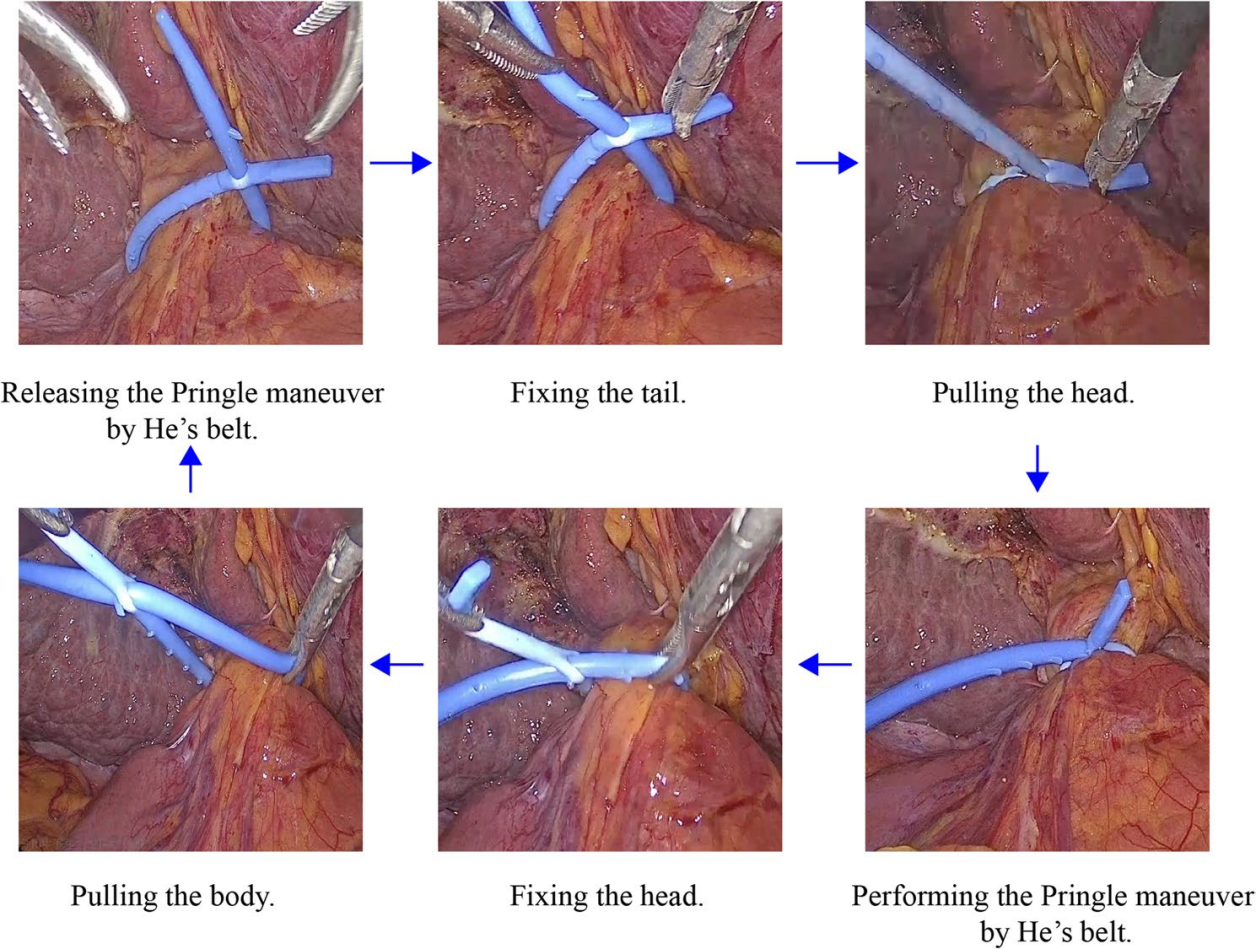
The procedure of performing intermittent Pringle maneuver by He’s belt

He’s belt is a silicone belt with a length of approximately 12 cm and a diameter of 3 mm, consisting of four parts: head, neck, body, and tail.(Fig. 1A) When it is necessary to clamp the hepatoduodenal ligament, the conical head and body are pulled out through the small tail hole, making the He’s belt become a ring structure that is not easy to slip off.(Fig. 1B) During the clamping period, one pair of cogs in the neck and eight pairs of cogs in the body are not easily slipped out or into the small hole due to cogs’ increased diameter, which ensures relatively constant and adjustable pressure of inflow occlusion. The procedure for presetting He’s belt is as follows: (Fig. 2) To start with, the tip of a laparoscopic forcep A is inserted into the tail hole of the He’s belt. Next, He’s belt is carried into the abdominal cavity and is placed on the right side of hepatoduodenal ligament. Immediately thereafter, another laparoscopic forceps B pass through the lesser omentum and pass behind the hepatoduodenal ligament to clamp the body of the He’s belt. The body of He’s belt is carried and encircles the hepatoduodenal ligament. Finally, the laparoscopic forceps A clamp the head and pull the head and body out of the hole, making the He’s belt become a ring structure in shape. The procedure of performing intermittent PM is as Fig. 3. When performing the Pringle maneuver, the tail is fixed, and the head is pulled to tighten the ring. And it is easy to adjust the pressure by pulling the cogs of the bodyout or into the hole. While releasing the Pringle maneuver, the head is fixed and the body is being pulled to loosen the ring.

### Patients

Between January 2024 and June 2024, continuous patients diagnosed with hepatolithiasis, benign liver tumors, primary liver cancer, or liver metastases, who were conducted laparoscopic hepatectomy and clamped hepatoduodenal ligament by He’s belt during operation, at the Guangdong Provincial Hospital of Chinese Medicine were retrospectively collected and reviewed for eligibility. Histopathological diagnosis of benign liver tumors, primary liver cancer, or liver metastases was based on the World Health Organization criteria.

The patient’s informed consent and confidentiality of information were obtained before the treatment. The retrospective analysis was approved by the Clinical Research Ethics Committee of Guangdong Provincial Hospital of Chinese Medicine (No. ZE2025-042-01). The Declaration of Helsinki on biomedical research involving human participants has also been followed.

### Statistical analysis

All the continuous values were expressed as the median (range or interquartile range) and categorical data were expressed as numbers or frequency. Comparison between groups was examined using a non-parametric test (Wilcoxon Signed Rank Test) for the continuous parameters and the χ2 test for the categorical parameters. All statistical analyses were performed with R version 4.0.2 (https://www.r-project.org/). Statistical significance was defined as *P* < 0.05.

## Result

### Patient demographics

This research included 47 patients who underwent laparoscopic liver resection and were clamped hepatoduodenal ligament by the He’s belt during operation. The clinical characteristics of patients are shown in Table 1. Thirty-three male and 14 female patients with a median age of 59 years old (range from 24 to 78) were enrolled. All patients had normal liver function before resection, all of patients were classified as Child A.

**Table 1:** Clinical characteristics of patients

Variables	*N* = 47
Male/Female	33/14 (70.2/29.8)
Ages (y), median, range	59.0 (24, 780)
Liver disease	
Primary liver cancer	28 (59.6)
Benign focal hepatic lesions	13 (27.7)
Liver metastases	3 (6.4)
Hepatolithiasis	3 (6.4)
Child Pugh Class (A/B)	50/0

### Perioperative outcomes

Among these 47 patients, 31/47 of patients were conducted anatomic liver resection. The median operative time was 121 min (IQR, 100–180). The median Pringle time was 21 min(IQR, 15.5–33.5). The median blood loss was 50 ml (IQR, 30–200). None of the patients required intraoperative blood transfusion. (Table 2)

**Table 2:** Operative characteristics and postoperative outcomes

Variables	*N* = 47
Type of hepatectomy	
Subsegment or Segment hepatectomy	17 (36.2)
Multisegment hepaectomy	24 (51.1)
Hemihepatectomy	6 (12.8)
Anatomic/non-anatomic	31/16
Operative time (min), median, IQR	121(100–180)
Pringle time (min), median, IQR	21 (15.5–33.5)
Estimated blood loss (ml), median, IQR	50 (30–200)
Intraoperative blood transfusion (no/yes)	47/0

## Discussion

LH is a safe and minimally invasive hepatectomy technique, with low mortality and morbidity rates [10]. Additionally, LH is comparable to open hepatectomy regarding long-term oncological benefits [11]. The Pringle maneuver has been successfully applied in laparoscopic hepatectomy, offering several advantages, including reduced blood loss, decreased need for perioperative blood transfusions, and a clearer operative field [6]. Many authors have introduced their PM technique during minimally invasive liver resection. Huang’s Loop or modified Huang’s Loop, an intracorporeal technique, has the benefit of not requiring an extra port comparable with the extracorporeal technique [7, 12]. However, Huang’s Loop also has some limitations. When performing and releasing the Pringle maneuver using Huang’s Loop, additional hemoclips were usually required. Herein, we introduced a PM technique by He’s belt, which was designed on the base of Huang’s Loop.

Compared to Huang’s Loop and other existing methods, He’s belt addresses these limitations through its compact design, ease of use, and elimination of the need for additional instruments such as hemoclips. When applying the extracorporeal PM methods, an additional trocar port would been puncturing and body damage would been increased [13]. Unlike extracorporeal methods, He’s belt eliminates the need for extra trocars, reducing bodily trauma. Huang’s Loop is typically made from a 14-Foley urethral catheter with a length of 15 cm, which may obstruct operative field of vision during surgery. Nevertheless, He’s belt is a small silicone tube with a length of approximately 12 cm and a diameter of 3 mm and can be easily rotated, therefore it rarely obstructs operative field [7]. When performing the Huang’s loop, one hemoclip was needed to prevent the loop structure loosing. While releasing the Huang’s loop, hemoclip was removed or been burned and cut by ultrasound knife, which may leading to thermal burn. However, He’s belt does not require additional hemoclips because the eight pairs of cogs in the body prevent slipping through the hole. Moreover, the ring structure formed by He’s belt can be performed and released repeatedly.

PM method often requires repeated application during complex hepatic resection procedures. Thus, elasticity and durability of He’s Belt were prioritized as key selection criteria for the optimal material, despite its original design as a single-use device. In the initial design phase, latex was evaluated due to its accessibility. However, its inadequate elasticity prevented the main body of He’s belt from traversing the tail hole, even after inner diameter enlargement. Consequently, silicone was selected as the final material, as it provided the necessary elasticity and durability for the device. Showed as Table 2, among 47 surgical procedures using new He’s belts (one per case), the median number of Pringle maneuvers was 2 (range 1–5) with a median total occlusion time of 21 min (range 6–67). Effective hepatic inflow control was achieved in all cases, including those requiring the maximum number of maneuvers (5 times) and prolonged occlusion duration (up to 67 min).

Complete occlusion of the inflow was achieved by performing PM method by He’s belt, whether the hepatoduodenal ligament was fat or thin. As shown in the Fig. 1A, B, there are 8 cogs in the body of He’s belt. The inner diameter of ring structure formed by He’s belt could be adjustable precisely by pulling out or in the cogs according the thickness of Hepatoduodenal ligament, which provided an appropriate pressure of inflow occlusion and avoided potential portal vein endothelium injury and portal vein thrombosis. Among the 47 patients conducted with LH in this study, the median Pringle time was 21 min. A small amount of blood loss benefited from the successful inflow occlusion by He’s belt.

There were several limitations for our study. He’s belt is contraindicated for individuals with a silicone allergy. Although medical-grade silicone has excellent low antigenicity and biocompatibility, published cases have documented allergic reactions to other medical-grade silicone products, such as breast implants and continuous positive airway pressure masks [14, 15]. Similar to all other hepatic inflow occlusion techniques, the use of the He’s belt is absolutely contraindicated in cases with portal vein thrombosis or tumor thrombus. Additionally, if fat debris is observed upon initial releasing the Pringle maneuver during surgery, suggesting inflammatory changes in the ligamentous tissue, we recommend discontinuing the Pringle maneuver (PM) to minimize further damage to the hepatoduodenal ligament. For patients requiring vascular skeletonization due to lymph node metastasis in the hepatoduodenal ligament, PM method by He’s belt would not be the preferred option. Interesting, our clinical experience observed that obesity does not contraindicate the use of He’s belt, as the thickness of the hepatoduodenal ligament does not exhibit direct proportionality with BMI. As shown in Fig. 1A and B, the maximum circumference of the loop formed by He’s belt measures approximately 9 cm, ensuring that even in obese patients, the belt possesses sufficient length to fully encircle the hepatoduodenal ligament. Moreover, the present study was retrospective, and observational within a single center, a prospective, multicenter study should be designed and performed to further valid the effect of PM method by He’s belt.

## Conclusion

For laparoscopic liver resection requiring inflow occlusion, the Pringle maneuver using He’s belt is a safe, effective, convenient, and self-adjustable method.

## Data Availability

The data that support the findings of this study are available from the corresponding author upon reasonable request.
